# Prevalence Estimates and Risk Factors of Anxiety among Healthcare Workers in Jordan over One Year of the COVID-19 Pandemic: A Cross-Sectional Study

**DOI:** 10.3390/ijerph19052615

**Published:** 2022-02-24

**Authors:** Ahmed Yassin, Abdel-Hameed Al-Mistarehi, Khalid El-Salem, Reema A. Karasneh, Sayer Al-Azzam, Aref A. Qarqash, Aws G. Khasawneh, Anas M. Zein Alaabdin, Ola Soudah

**Affiliations:** 1 Department of Neurology, Faculty of Medicine, Jordan University of Science and Technology, Irbid 22110, Jordan; kelsalem@just.edu.jo; 2 Department of Public Health and Family Medicine, Faculty of Medicine, Jordan University of Science and Technology, Irbid 22110, Jordan; awalmistarehi18@med.just.edu.jo (A.-H.A.-M.); azainalabdin@yahoo.com (A.M.Z.A.); 3 Department of Basic Medical Sciences, Faculty of Medicine, Yarmouk University, Irbid 21163, Jordan; reema.karasneh@yu.edu.jo (R.A.K.); ola.soudah@yu.edu.jo (O.S.); 4 Department of Clinical Pharmacy, Jordan University of Science and Technology, Irbid 22110, Jordan; salazzam@just.edu.jo; 5 Faculty of Medicine, Jordan University of Science and Technology, Irbid 22110, Jordan; aaqarqash1819@med.just.edu.jo; 6 Department of Psychiatry, Faculty of Medicine, Jordan University of Science and Technology, Irbid 22110, Jordan; agkhasawneh3@just.edu.jo

**Keywords:** COVID-19, anxiety, healthcare workers, health providers, physicians, one-year

## Abstract

This study investigates the changes in prevalence estimates, severity, and risk factors of anxiety among healthcare workers (HCWs) over the first year of the COVID-19 pandemic. A survey was distributed among HCWs using snowball sampling, collecting their socio-demographics, occupation, and anxiety symptoms as measured by the Generalized Anxiety Disorder-7 (GAD-7) scale. It was distributed one month after the pandemic’s onset in Jordan between 15 and 30 April 2020 (onset group) and after one year between 15 and 30 March 2021 (one-year group). A total of 422 HCWs were included (211 in each group). The one-year group reported a higher risk of GAD (30.8% vs. 16.6%; *p* = 0.001), a higher mean (SD) GAD-7 score (7.94 (5.29) vs. 6.15 (4.15); *p* < 0.001), and more severe symptoms (*p* = 0.003). Univariate analyses showed that participants who were younger, women, unmarried, had lower monthly incomes, underwent testing for COVID-19, had higher contact with COVID-19 patients, did not receive special education, and were unsatisfied with the institutional COVID-19 preparedness scored higher on the GAD-7 scale and had more severe symptoms than their counterparts in both groups. Unlike the onset group, occupation as a physician, COVID-19 infection history, and perception of remarkable changes in work were associated with higher anxiety scores and severity among the one-year group. The COVID-19 vaccine was a relative protective action. Logistic regression analyses showed that the female gender was a risk factor for developing GAD at the pandemic onset, while poor satisfaction with institutional preparedness was a significant GAD risk factor in the one-year group. Low monthly income and lack of special education were the shared risk factors for GAD in both groups. This study reveals a significant rise in anxiety among HCWs over a year of the COVID-19 pandemic and shows the vulnerable sub-groups who likely need psychological interventions.

## 1. Introduction

Severe Acute Respiratory Syndrome Coronavirus 2 (SARS-CoV-2), the cause of Coronavirus Disease 2019 (COVID-19), continues to viciously spread across the world. According to the Johns Hopkins Coronavirus Resource Center, as of 1 January 2022, 222 nations have been affected by the COVID-19 pandemic, with more than 288 million confirmed cases and over 5.4 million deaths globally [1]. The COVID-19 pandemic and its associated precautionary measures have negatively impacted life in all possible ways [2,3,4,5,6]. In addition to the respiratory manifestation that COVID-19 mainly presents with, other manifestations and complications of the infection are vast and could include cardiovascular, thromboembolic, and neurological ones [7,8,9,10,11]. In addition, COVID-19 has considerable psychological effects on patients and the general public as a whole [12,13,14]. A Swedish cross-sectional study conducted early in the pandemic found that 28.3% of the general population reported clinically significant anxiety levels [13].

Healthcare workers (HCWs), who are continuously exposed to the infection, have been significantly affected. Studies have shown that HCWs, including emergency HCWs and those engaged in the direct care of COVID-19 patients, have experienced significant and persistent stress, which is likely related to their perceived fear of acquiring and spreading the infection [15,16]. Such stress has resulted in significantly high levels of secondary trauma, burnout, tension, difficulties in teamwork, irritable mood, and physical and mental fatigue [15,16]. These studies emphasized the need for developing coping mechanisms, hardiness, and resilience to alleviate and endure such stress-related complications and the importance of preparing preventive strategies for future pandemics [15,16]. Vagni et al. (2020) found that female HCWs have significantly higher physical, emotional, and COVID-19 stress than males, with no gender difference in coping mechanisms and secondary trauma [15]. Other factors having a predictive effect on the stress and well-being of HCWs include age, professional role, and exposure to COVID-19 patients [15,16,17]. Other studies reported that stress and anxiety levels are proportional to the risk of infection, with higher perception levels in HCWs living in outbreak areas [18]. Death anxiety during the pandemic has been linked to COVID-19-related anxiety, and it was found to be mainly related to the HCWs’ worry about fatal or severe consequences of COVID-19 [18,19].

Many studies, including systematic reviews and meta-analyses, found high prevalence rates of anxiety and depression among HCWs during the COVID-19 pandemic [20,21]. A study on the general population across the United States of America (USA) found that employment as HCW was a significant risk factor for anxiety [22]. This could be attributed to their perceived risk of acquiring the infection upon direct contact with suspected or confirmed cases [23]. Other contributing factors to anxiety include the increase in their workload; their worries of transmitting the infection to other patients, colleagues, loved ones, and family members; the negative feelings that progressively build up as HCWs see the suffering of patients and their families; the periods of lockdown and movement restriction; and the harmful effects of the pandemic on their social support system [23,24,25,26]. Thus, HCWs are vulnerable to this psychopathological stress, increasing symptoms of depression and anxiety [27,28,29,30,31].

**Figure 1:**
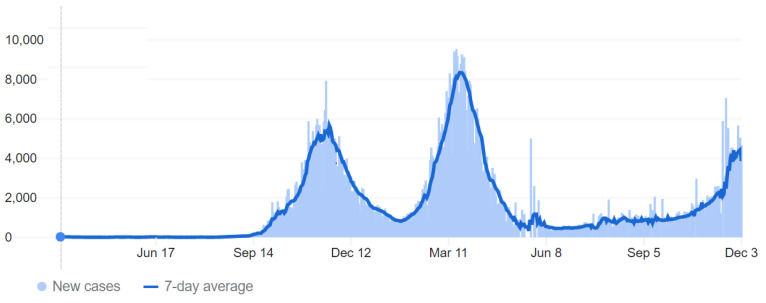
Coronavirus Disease 2019 (COVID-19) daily and weekly new confirmed cases in Jordan over 2020–2021. The first case was confirmed on 2 March 2020; the first peak was around November 2020, and the second one was around April 2020. The figure was adapted from COVID-19 Dashboard by the Center for Systems Science and Engineering (CSSE) at Johns Hopkins University (JHU) [1,34].

On 2 March 2020, the first COVID-19 case was confirmed in Jordan, with no new reported cases of COVID-19 until 15 March, when 11 new cases had been tested positive for COVID-19, followed by a rise in cases in the following days and weeks [8,32]. Accordingly, on 17 March, the government enforced a complete lockdown for three months, resulting in a relatively limited number of cases during this period. A few months after lockdown’s end, around October to November 2020, the number of cases started to rise significantly, reaching the “first peak”. This pushed the government to impose partial restrictions on people’s movement, including a daily curfew after 9 pm and a complete curfew on Fridays (the official weekly holiday in Jordan). The numbers became relatively under control towards the end of 2020, and restrictions were loosened. However, later in February and March 2021, cases started to rise again, reaching the “second peak”, pushing the government to tighten the restrictions again [1,33,34] (Figure 1).

The perception and workload of HCWs changed overtime during the pandemic. In the beginning, there was a low number of cases but a significant mystery and unpredictability around the disease in terms of morbidity, mortality, how long it will last, and whether effective vaccines and treatments would be developed to control the infection. One year after that, a significant rise in the cumulative number of COVID-19 cases occurred with a progressive increase in the disease burden that particularly impacted HCWs. With that, significant changes happened, including the increase in the familiarity of HCWs with the disease precautions, handling patients presenting with suspected or confirmed COVID-19 infections, the emergence and use of multiple vaccines, along with the development of management guidelines and protocols. Considering such changes between the early period of the pandemic and one year after its onset, changes in anxiety prevalence rates and risk factors among HCWs were expected and worth investigating. In addition, this article deals with the reaction to a different social and cultural reality than mostly reported in the literature, for example, the USA and Europe. Thus, this study aims to figure out the prevalence rates, severity degrees, and risk factors of anxiety symptoms among HCWs one month after the first COVID-19 case was recorded in Jordan and compare that with the numbers one year after (during the second peak). To our knowledge, this study is one of the first to assess the trend of anxiety symptoms among HCWs over a one-year period of the COVID-19 pandemic. The ultimate goal is to recommend interventions to alleviate anxiety symptoms, particularly for vulnerable sub-groups, and potentially prevent these symptoms from occurring if similar health crises occur in the future. Such recommendations would be directed to the attention of health care providers and health administration at institutional, national, and even international levels.

## 2. Materials and Methods

### 2.1. Study Design, Population, and Ethical Approval

A cross-sectional study was conducted through two stages. The survey was conducted online using the Google Form tool. The survey was firstly distributed between 15 and 30 April 2020, approximately one month after the onset of COVID-19 in Jordan, and this group of respondents was named the “onset” group. Then, the same questionnaire was distributed for a second time between the 15 and 30 March 2021, one year after the onset of COVID-19 in Jordan, during the second peak. The group of respondents in this round was named the “one-year” group. Participants were eligible if they were HCWs, living and working in Jordan, aged 18 years or older, and had internet access. The researchers shared the e-survey link via social media platforms, mainly WhatsApp, and a snowball sampling was performed by asking the participants to distribute the e-survey further to their peers. The e-surveys were distributed between HCWs from all sectors of healthcare in Jordan, including university hospitals, Ministry of Health (MOH) hospitals, military hospitals, and private hospitals. On clicking the received link, the respondents would be directed to the informed consent form, which includes a short description of the objectives and design of the study followed by a consent question plea. If they agree to participate, they will be directed to the e-survey questions. If they refuse to participate, the form will terminate. Participants could terminate the e-survey at any time desired. The survey was anonymous, and information confidentiality was assured. Participants did not receive any rewards or compensation for their participation in this study.

The study design was ethically approved by the Institutional Review Board (IRB) of the research and ethics committee at Jordan University of Science and Technology, Irbid, Jordan (IRB number of 106/132/2020). This study was conducted following the 1975 Helsinki declaration, as revised in 2008 and its later amendments or comparable ethical standards.

### 2.2. Survey Instruments

The questionnaire consisted of three sections: socio-demographic characteristics, occupational situation, and anxiety scale. Socio-demographic characteristics included age, gender, area of residence, marital status (married, single, widowed, or divorced), whether they were living with elderly of 65 years or older, personal history of undergoing COVID-19 testing, personal history of COVID-19 infection, and whether they needed hospitalization if they had been infected. The history of receiving the COVID-19 vaccine was investigated only in the one-year group, as the vaccine was unavailable for the first 10 months of the COVID-19 pandemic [35].

Questions about occupation included asking about working position (physician, nurse, pharmacist, technician), monthly income in Jordanian Dinar (JD), whether they were in direct contact with confirmed or suspected COVID-19 individuals and samples during their work (yes or no), the estimated number of confirmed or suspected COVID-19 individuals and samples that participants dealt with, and whether they received a special education to deal with COVID-19 patients (yes or no). Participants’ perceptions of the level of contact with COVID-19 patients and samples were assessed using a 5-point Likert scale, ranging from “1 = low level of contact” to “5 = high level of contact”. Furthermore, participant’s evaluation of the institution’s preparedness to deal with COVID-19 patients was assessed using a 6-point Likert scale, ranging from “very bad” to “excellent”. In addition, the perceived level of change in work schedule and intensity due to the COVID-19 pandemic was investigated with response options of “no perceived changes/a little/some/much/very much”.

The last part of the questionnaire assessed anxiety symptoms experienced by the participants using the 7-item Generalized Anxiety Disorder (GAD-7), an efficient, reliable, and validated tool for screening GAD and assessing its severity [36,37,38,39,40]. The GAD-7 scale consists of seven items, based on the Diagnostic and Statistical Manual of Mental Disorders, fourth edition (DSM-IV) diagnostic criteria for GAD, asking how often the individual was bothered by each symptom during the preceding two weeks. Response options were “not at all”, “several days”, “more than half the days”, and “nearly every day” and were scored 0, 1, 2, and 3, respectively. Then, the scores were summed for each participant to obtain the total score (range, 0–21). At the cutoff point of 10 for the high probability of GAD diagnosis, the GAD-7 scale has a sensitivity of 89% and a specificity of 82% [36]. Thus, participants with a total score of ≥10 on the GAD-7 scale were categorized into the highly probable GAD group. The anxiety severity was categorized based on the total score of GAD-7 into normal (0–4), mild (5–9), moderate (10–14), and severe (15–21) anxiety [36]. In our study, the Cronbach’s α of the GAD-7 scale items was 0.904.

The questionnaire validity was checked by a pilot study that included 20 random HCWs who assessed the questionnaire’s clarity, and no significant modifications were required.

### 2.3. Statistical Analysis

All data analyses were performed using the IBM Statistical Package for the Social Sciences (SPSS) software for Windows, version 25.0. Continuous variables, including age, perceived level of contact with COVID-19 patients, and GAD-7 scale total scores, were presented as mean ± standard deviation (m ± SD) after checking and verifying the normality distribution of the dataset. The age variable was further presented as a categorical variable with four groups, based on the interquartile ranges, including 23–27, 28–31, 32–39, and ≥40 years. Descriptive statistics were conducted to calculate the frequencies and percentages for the categorical variables. Internal consistency reliability was measured using Cronbach’s α for the GAD-7 scale.

The differences between onset and one-year groups were analyzed using a chi-square test for categorical variables, including socio-demographic, occupational characteristics, and severity categories of anxiety symptoms. In contrast, parametric tests, including Student’s t-test or one-way ANOVA, were used for continuous variables after confirming the normality distribution of their data, including GAD-7 scale total scores and perceived level of contact with COVID-19. In addition, we investigated the differences in the GAD-7 scale total scores among each sample separately using Student’s t-test or one-way ANOVA. The differences in the severity categories of anxiety among each sample were also assessed using a chi-square test.

Binary logistic regression analyses were used to estimate the Odds Ratio (OR) and 95% Confidence Interval (95% CI) for GAD risk factors among each sample of HCWs. The dependent variable was the high probable GAD diagnosis identified by a total GAD-7 score of ≥10; thus, it included moderate and severe anxiety categories [36]. The age, gender, marital status, living with the elderly, occupation, monthly income, COVID-19 vaccination, previous testing, previous infection, direct contact with COVID-19 patients and samples during work, receiving a special education to deal with COVID-19 patients, evaluation of institution COVID-19 preparedness, and perceived changes in work schedule and intensity due to the COVID-19 pandemic were included as independent explanatory variables. Model selection using the stepwise backward approach with a cutoff *p*-value of 0.2 was used to select the final, most parsimonious model. The independent variables in the last model were checked for multicollinearity using variance inflation factor (VIF). Statistical significance was considered at a *p*-value of ≤0.05.

## 3. Results

### 3.1. Participation Rate

**Figure 2:**
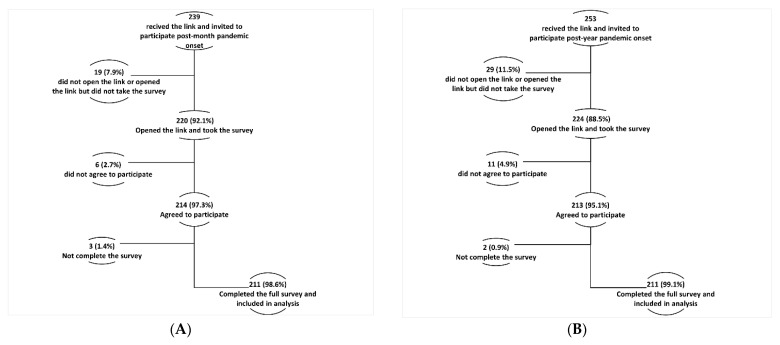
(**A**) Study participants flow chart post-month of COVID-19 pandemic onset. (**B**) Study participants flow chart after one year of COVID-19 pandemic.

In this study, among the 494 HCWs invited to participate (239 in the first round and 253 in the second round), 427 respondents initiated the e-survey, with a participation rate of 86.4%. Of the respondents, 422 (98.8%) completed the e-survey items and were included in the final sample (Figure 2A,B).

### 3.2. Total Cohort’s Socio-Demographic Characteristics

The participants’ age ranged from 23 to 73 years with a mean (SD) of 35.3 (9.9) years, and 71.3% were men. Of the total cohort, 254 (60.2%) were married, and 168 (39.8%) were single, widowed, or divorced. Most participants (*n* = 344, 81.5%) were physicians, while 78 (18.5%) were nurses, pharmacists, or technicians. More than half of the participants (58.1%) reported a low monthly income with less than JOD 1000.

### 3.3. The Onset and One-Year Samples’ Characteristics

Each group of the two samples included 211 participants, representing 50% of the total cohort. The two groups matched in the sample size, age, gender, marital status, occupation, and monthly income (*p* > 0.05 for each). Among the onset sample, the age ranged between 24 and 70 years with a mean (SD) of 34.7 (9.3), 73.0% were male participants, 62.6% were married, 77.7% were physicians, and 62.5% reported a low monthly income. Among the one-year group, the age ranged from 23 to 73 years with a mean (SD) of 35.8 (10.5), 69.7% were male participants, 57.8% were married, 85.3% were physicians, and 53.6% reported a low monthly income. Table 1 shows the participants’ socio-demographic and occupational characteristics in the total cohort and its subgroups.

Within the one-year group, the proportions of HCWs who were tested for (87.2%) or infected with (46.0%) COVID-19 were significantly higher than that of the onset group (23.2% and 0.5%, respectively) (*p* < 0.001 for each). In addition, the HCWs’ contact with COVID-19 patients and samples in the one-year group was significantly higher than the rate in the onset group (*p* < 0.001). Most participants of the two samples did not receive a special education to deal with COVID-19 patients, with no significant differences between the two groups. More participants in the one-year sample were unsatisfied with the institutional preparedness to deal with COVID-19 patients than the onset sample. There was no significant difference in the perception of changes in work schedule and intensity among the two groups (*p* = 0.474).

### 3.4. Trends of Anxiety Symptoms among HCWs over a Year of COVID-19 Pandemic

In the total cohort, a high possibility of GAD, as identified with the GAD-7 score of ≥10, was observed in 100 (23.7%) of participants, with a mean (SD) GAD-7 score of 7.1 (4.8). Moreover, the anxiety symptoms were mild in 190 (45.0%), moderate in 58 (13.7%), and severe in 42 (10.0%) participants of the total cohort.

**Figure 3:**
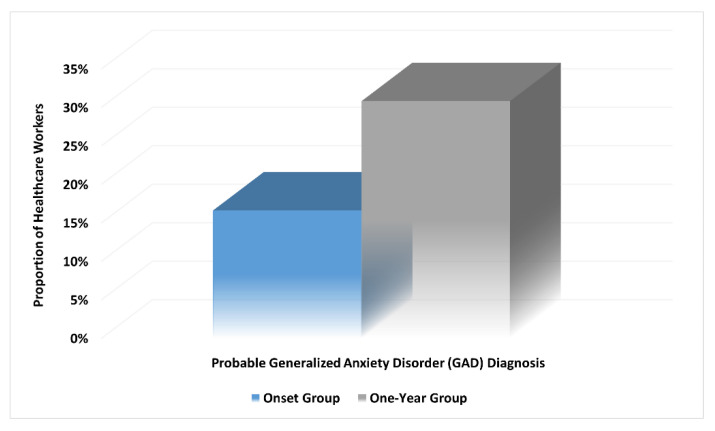
Prevalence trends of healthcare workers with possible Generalized Anxiety Disorder (GAD) diagnosis over one year of the COVID-19 pandemic (*p* = 0.001).

For the one-year group, 65 (30.8%) participants had a high probability of GAD, which is significantly higher than the onset group (*n* = 35, 16.6%) (unadjusted OR of 2.239; 95% CI, 1.405–3.567; *p* = 0.001) (Figure 3). Moreover, the mean (SD) scores of the GAD-7 scale for anxiety were significantly higher among the one-year group (7.94 (5.29)) compared to the onset group (6.15 (4.15)) with a mean difference of 1.79 (t(420) = 3.86, *p* < 0.001). In addition, more participants from the one-year sample fell in the moderate to severe anxiety categories compared with the onset group (*p* = 0.003). Table 2 shows the scores and severity categories of anxiety symptoms among HCWs in the total cohort and the onset and one-year groups.

### 3.5. Factors Associated with Anxiety Symptoms in the Onset Group

In the onset sample, younger, women, and unmarried participants had significantly higher anxiety scores than their counterparts (Table 3). Data from this sample showed a trend of significantly decreasing anxiety scores with increasing monthly income. In addition, HCWs who had been tested for COVID-19 reported higher mean scores on the GAD-7 scale (7.63 (4.59)) than those who had not been tested (5.70 (3.91)) (*p* = 0.004). Similarly, higher anxiety mean scores were observed among HCWs with direct contact with COVID-19 patients and samples than those who did not report such contact (7.75 (4.89) vs. 5.63 (3.75), *p* = 0.001). HCWs who reported not receiving a special COVID-19 education had significantly higher scores on the GAD-7 scale than those who received such education (6.74 (4.18) vs. 4.63 (3.69), *p* = 0.001). Lastly, lower satisfaction with institutional COVID-19 preparedness was significantly associated with higher anxiety scores (Table 3).

Regarding the severity categories of anxiety, similar to the previous findings based on GAD-7 mean scores, HCWs who were younger, women, unmarried, had a lower monthly income, underwent testing for SARS-CoV-2 infection, had high contact with COVID-19 patients and samples, reported not receiving special COVID-19 education, or were unsatisfied with the institutional preparedness had higher severity of anxiety symptoms, based on severity categories, than their counterparts (*p* < 0.05) (Table 3).

However, living with the elderly, occupation, and perceived changes in work schedule or intensity were not significantly associated with anxiety scores or severity categories among the onset group (*p* > 0.05).

### 3.6. Factors Associated with Anxiety Symptoms in the One-Year Group

Although the one-year participants had higher scores and severity levels of anxiety than the onset group, the distribution of anxiety symptoms within socio-demographic and occupational categories was almost consistent with patterns observed in the onset group. Higher anxiety, based on mean scores and severity categories, were observed again among participants who were younger, women, unmarried, had lower monthly income, reported previous testing for COVID-19, had high direct contact with COVID-19 patients and samples, did not receive special COVID-19 education, and were unsatisfied with the institutional COVID-19 preparedness (Table 4).

**Figure 4:**
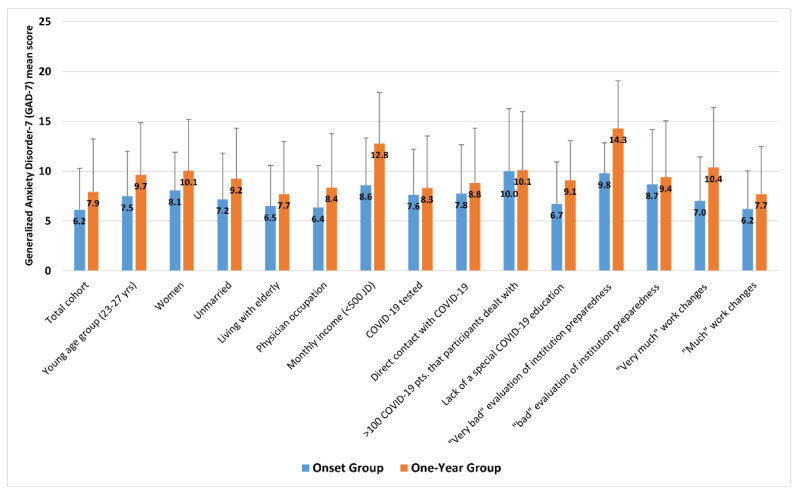
Differences of Generalized Anxiety Disorder-7 (GAD-7) mean scores by participants’ characteristics between the onset and one-year groups.

However, unlike the onset group, more participants’ characteristics were significantly associated with anxiety scores and symptoms in the one-year group. For occupation, physicians in the one-year group had significantly higher scores of anxiety (8.37 (5.43)) than other HCWs (5.45 (3.54)) (*p* = 0.004), as well as physicians in the onset group (6.36 (4.20)) (*p* < 0.001). Moreover, unlike the onset group, physicians in the one-year group experienced significantly more severe anxiety symptoms, based on anxiety categories, than other HCWs (*p* = 0.004). In addition, unlike the onset group participants, data from the one-year sample indicated that participants who perceived more remarkable changes in work schedule and intensity due to the pandemic had a more significant burden of anxiety symptoms (*p* < 0.001). As shown in Figure 4, the one-year group reported significantly higher GAD-7 scores than the onset group among the vast majority of socio-demographic and occupational characteristics of participants.

Among the one-year group, approximately half of the participants (46.0%) reported a history of COVID-19 infection, and they had significantly higher anxiety, based on GAD-7 scores and anxiety severity, than those who had not been infected (*p* < 0.05). Although 71.6% of HCWs became vaccinated against COVID-19 one year after the pandemic’s onset, there were no significant differences in the anxiety mean (SD) scores between vaccinated (7.75 (5.32)) and non-vaccinated participants (8.01 (5.30)) (*p* = 0.745). Like in the onset sample, living with the elderly was insignificant for anxiety (scores and severity categories) among one-year participants (*p* > 0.05).

### 3.7. Risk Factors for Generalized Anxiety Disorder (GAD) among HCWs

Binary logistic regression analyses showed that, after controlling for the confounders, the lack of special education on how to deal with COVID-19 patients was a significant independent risk factor for developing clinically significant levels of anxiety symptoms among the onset group (OR, 3.25; 95% CI 1.123–9.378; *p* = 0.030) and the one-year group (OR, 6.05; 95% CI 2.394–15.296; *p* < 0.001). In addition, low monthly income was another significant GAD risk factor within both groups (Table 5). The female gender was a significant independent risk factor for developing GAD in the onset group (OR, 3.22; 95% CI 1.440–7.218; *p* = 0.004). On the other hand, grading the institutional COVID-19 preparedness as “very bad” was a significant independent risk factor for developing anxiety symptoms (OR, 8.72; 95% CI 1.215–62.523; *p* = 0.031) among the one-year group participants. Lastly, among both onset and one-year samples, HCWs who reported direct contact with COVID-19 patients and samples were twice more likely to develop anxiety symptoms than those with no such contact. However, this factor did not reach the statistical significance cutoff value of ≤0.05 (Table 5).

## 4. Discussion

To our knowledge, this study is one of the first to investigate the change trends in prevalence rates, severity, and risk factors of anxiety among HCWs over a year of the COVID-19 pandemic. Our study showed a high proportion of HCWs (23.7%) manifesting anxiety symptoms with a significant increase in the prevalence rate over the first year of the pandemic in Jordan (16.6% at onset vs. 30.8% after one year), with a significant increase in the GAD-7 mean scores and the percentage of participants in the moderate and severe anxiety categories. The change in the risk factors for anxiety symptoms was not impressive. Upon univariate analysis, HCWs who were younger, females, unmarried, had lower monthly incomes, underwent testing for COVID-19, had direct contact with COVID-19 patients and samples, did not receive a special COVID-19 education, or were unsatisfied with the institutional preparedness reported higher anxiety scores and had more severe symptoms than their counterparts in both the onset and one-year groups. Living with the elderly was insignificant for anxiety scores and severity levels among the onset and one-year samples. However, unlike the onset group, occupation as a physician and perceived remarkable changes in work schedule or intensity were associated with a higher risk for anxiety among the one-year group. As expected, the one-year group had much higher rates of COVID-19 infection, which were associated with significantly higher anxiety scores and severity. A COVID-19 vaccine was received by two-thirds of HCWs one year after the onset of the COVID-19 pandemic and was considered a relative, but insignificant, protective action. Using binary logistic regression, low monthly income and not receiving special COVID-19 education were shared risk factors for developing GAD among the two groups. The female gender was a significant GAD risk factor in the onset group, while poor satisfaction with the institutional COVID-19 preparedness was a significant GAD risk factor in the one-year group.

### 4.1. Anxiety among HCWs

Previous studies showed a significant but unspoken high anxiety risk among HCWs [41,42,43]. A multicentric survey-based study in China using the GAD-7 scale on 1257 HCWs found that 45% manifested anxiety symptoms [41]. A study in northeast Italy found that 50% of HCWs showed clinically relevant anxiety symptoms [42]. A multicenter, cross-sectional study on HCWs in Ghana conducted from 11 July to 12 August 2020 found that 28% of participants had anxiety [43]. None of these studies trended anxiety scores and severity among HCWs over a long duration as our study investigated.

### 4.2. Anxiety Risk Factors

#### 4.2.1. Shared Risk Factors between the Onset and One-Year Groups

Our study showed that younger age and unmarried status were persistent risk factors for anxiety over the year of this study. Similar to our findings, previous studies have shown that younger age was a significant predictor of anxiety during the COVID-19 pandemic [13]. The movement restrictions and social isolation associated with the pandemic could have resulted in more anxiety symptoms [44,45]. The recurrent periods of lockdown and restrictions on people’s movement throughout the first year had a persistent negative effect on people, mainly the younger unmarried ones, including HCWs, who are by nature more socially active than older married people. Such limitations in social life could have contributed to their persistent anxiety over time. In addition, the literature reported a supportive social system as a protective factor against anxiety during the COVID-19 pandemic [13,46]. A French study showed that loneliness increased the risk of anxiety during COVID-19 lockdowns [4].

The testing for COVID-19 was a risk factor for developing anxiety symptoms among both groups. Testing for COVID-19 at the beginning of the pandemic was associated with amplified fear of getting a positive result with its associated consequences, including movement and work restrictions and the fear of the not-yet-known morbidity and mortality outcomes COVID-19 in the early period of the pandemic. On the other hand, more frequent testing for COVID-19 over a year of the pandemic could have resulted from frequent and more prolonged contact with patients with confirmed or suspected infections, which could be the reason behind the persistent significance of testing as a risk factor for anxiety one year after the pandemic’s onset.

Our findings also showed that HCWs who had direct contact with COVID-19 patients or samples had a higher risk for anxiety in both groups. Previous studies showed that HCWs directly engaged with COVID-19 patients had a higher risk and more severe degrees of anxiety [41,42,46,47]. This might reflect the amplified perception of risk for acquiring and transmitting infection on contact with COVID-19 patients and samples at the beginning of the pandemic and the persistent fear of acquiring the infection over time as cases increased exponentially in number. W. Lu et al. (2020) reported that frontline medical workers with close contact with infected COVID-19 patients have higher fear, anxiety, and depression scores than administrative staff [27].

Using binary logistic regression, lower monthly income was a significant risk factor for GAD in the onset and one-year groups. This observation indicates that limited income became more burdening and resulted in more anxiety symptoms over time. Many studies showed a similar negative impact of low economic status or losing a job on the psychological immunity of adults against stressful times during COVID-19 [4,13]. Financial support, on the other hand, such as the government’s tax-free salary relief, was shown to reduce the adverse psychological effects of the pandemic [43].

There was no significant difference in the percentages of HCWs who received a special education to deal with COVID-19 between the onset and one-year groups, but this factor was significantly associated with higher anxiety scores after one year of the pandemic than its onset time. However, using binary logistic regression, the lack of such education was another significant risk factor for GAD in both groups. This finding indicates that lacking knowledge about a new health crisis has a persistent similar negative impact on the psychology of HCWs over time.

#### 4.2.2. Risk Factors at Onset of the Pandemic

Using binary logistic regression, female gender was a significant risk factor for developing GAD in the onset group only. This finding is concordant with previous studies that indicated that females in general and female HCWs have a higher risk and more severe degrees of anxiety than males [4,41,42,46]. This could be attributed to the higher stress experienced by female HCWs at the onset of the pandemic [48]. This stress includes the added role of female HCWs in caring for their homes and children in addition to their work responsibilities, the higher worry women have about their health and the health of their families, and their higher sensitivity towards a new health crisis [4,49,50]. These factors were maximal at the onset of such unpredictable and non-previously experienced stress and likely eased up with time [51].

#### 4.2.3. Risk Factors after One Year of the Pandemic

Among HCWs, physicians had the highest rates and severity scores of anxiety after one year of battling against COVID-19. This finding could be attributed to their responsibility for managing patients with COVID-19 and dealing with their morbidity and mortality. A recent systemic review reported high prevalence rates of anxiety among physicians (17% and 19.8%) [20]. A cross-sectional, web-based study investigating the mental health outcomes among HCWs during the COVID-19 pandemic in Italy found that general practitioners were more likely to report post-traumatic stress disorder (PTSD) than other HCWs [52].

There were no significant differences in the perception of change in work schedule and intensity among the two groups. However, the stress of change in work schedule and intensity resulted in higher anxiety symptoms over time. This finding emphasizes the importance of stress chronicity as a risk factor for developing anxiety symptoms. A study from the USA on the general population found that the prevalence of anxiety during the pandemic was statistically significantly higher among those who worked full time compared with part-timers and unemployed people [22].

One-year participants were less satisfied with the institutional preparedness to deal with COVID-19 patients than the onset group, which is likely an expected result of the increased burden of the pandemic over time. A “Very bad” evaluation for the institutional preparedness was, based on multivariate logistic regression, a significant risk factor for anxiety in the one-year group. In addition, HCWs who had been infected from the one-year group had significantly higher anxiety scores and more severe symptoms than those who had not. Such findings could be explained by the reported psychopathological effects of the virus in the literature, the infection-associated stress, and the psychotropic effects of treatments used in COVID-19 infection, such as hydroxychloroquine and corticosteroids [7,53,54,55,56,57,58,59]. Vaccination became routine across HCWs after one year of the COVID-19 pandemic, and although not statistically significant, COVID-19 vaccination was associated with lower anxiety mean (SD) scores among vaccinated participants than unvaccinated ones. This finding is concordant with a previous study that reported an association between receiving the first dose of the COVID-19 vaccine and decreased mental distress levels as measured by the four-item Patient Health Questionnaire (PHQ-4) [60].

#### 4.2.4. Strengths and Limitations of the Study

The study timeliness in investigating the change trends in prevalence rates, severity, and risk factors of anxiety among HCWs over the first year of the COVID-19 pandemic using snowball sampling is one of its strengths. In addition, this study obtained insights into anxiety burden among HCWs in an Eastern Mediterranean developing country. Thus, we tried to fill the literature gap regarding such issues outside Western countries. However, this study has a few limitations that should be mentioned. The sample size of participants was relatively small and the achieved representativeness was low, limiting our findings to the broader Jordanian population and other populations. Thus, the results are unlikely to be generalizable beyond the people who responded. The study was based on a cross-sectional design with inherent limitations that could affect the interpretation of the results. The study did not survey the same HCWs to figure out the exact trend changes in their anxiety scores, severity, and risk factors. This limitation, however, was compensated for by the fact that the onset and one-year groups matched in age, gender, marital status, occupation, and monthly income.

Additionally, web-based studies could not exclude the possibility of e-survey replication by the same individuals. In addition, we could not figure out how many participants from the first sample also participated in the second sample. This limitation could be attributed to the anonymous nature of the survey. Finally, most respondents were physicians and men, which despite being relatively similar in both onset and one-year surveys, makes the generalization of the results to all HCWs and particularly female HCWs less accurate. However, this can partly be explained by the fact that most of the HCWs in Jordan (70%) are males [61].

## 5. Conclusions

The study findings indicated that healthcare providers have a high prevalence of anxiety symptoms, which has increased, along with anxiety severity, over a one-year period during the COVID-19 pandemic. This necessitates swift mental healthcare interventions to this crucial population during the COVID-19 pandemic. Targeting vulnerable groups is also crucial for implementing these interventions. The distribution patterns of anxiety risk factors had not significantly changed over time. Persistently vulnerable HCWs included those who were younger, women, unmarried, had low monthly income, reported previous testing for COVID-19, had high contact with COVID-19 patients and samples, did not receive special COVID-19 education, and were unsatisfied with the institutional COVID-19 preparedness. After one year of the pandemic, the anxiety symptoms were significantly more intense and evident with these factors. In addition, an occupation as a physician, intense work schedules, and becoming infected with COVID-19 had higher anxiety scores and severity levels of anxiety symptoms after one year of the pandemic’s onset. An urgent need for healthcare officials to implement psychological interventions, strategies, and policies is suggested in order to promote mental health wellness among HCWs exposed to COVID-19 and other such vulnerable subgroups. The influence of socio-demographics and occupational situations of HCWs could be used to fine-tune the interventions.

## Figures and Tables

**Table 1:** Socio-demographic and occupational characteristics of the study participants in the total cohort and the onset and one-year subgroups.

Characteristic	Total Cohort, *n* = 422 *n* (%)	Onset Group, *n* = 211 *n* (%)	One-Year Group, *n* = 211 *n* (%)	*p*-Value
***Age, y ^®^***				
23–27	90 (21.3)	47 (22.3)	43 (20.4)	*0.761*
28–31	107 (25.4)	56 (26.5)	51 (24.2)	
32–39	118 (28.0)	59 (28.0)	59 (28.0)	
≥40	107 (25.4)	49 (23.2)	58 (27.5)	
***Gender***				
Male	301 (71.3)	154 (73.0)	147 (69.7)	*0.451*
Female	121 (28.7)	57 (27.0)	64 (30.3)	
***Marital status***				
Unmarried *	168 (39.8)	79 (37.4)	89 (42.2)	*0.320*
Married	254 (60.2)	132 (62.6)	122 (57.8)	
***Living with elderly of 65 years old or older***				
No	217 (51.4)	125 (59.2)	92 (43.6)	*0.001*
Yes	205 (48.6)	86 (40.8)	119 (56.4)	
***Occupation***				
Physician	344 (81.5)	164 (77.7)	180 (85.3)	*0.060*
Others ^†^	78 (18.5)	47 (22.3)	31 (14.7)	
***Monthly income, Jordanian Dinar (JD)***				
<500	56 (13.3)	25 (11.8)	31 (14.7)	*0.066*
500–1000	189 (44.8)	107 (50.7)	82 (38.9)	
1000–2000	66 (15.6)	33 (15.6)	33 (15.6)	
>2000	111 (26.3)	46 (21.8)	65 (30.8)	
***COVID-19 characteristics***				
Vaccinated against COVID-19 ^¥^	-	-	151 (71.6)	*-*
Tested for COVID-19	233 (55.2)	49 (23.2)	184 (87.2)	*<0.001*
Hx of COVID-19 infection	98 (23.2)	1 (0.5)	97 (46.0)	*<0.001*
Hx of hospital admission due to COVID-19 infection (% out of infected persons)	5 (5.1)	0 (0.0)	5 (5.2)	*-*
Direct contact with confirmed or suspected COVID-19 individuals or samples	197 (46.7)	52 (24.6)	145 (68.7)	*<0.001*
Perceived level of contact with COVID-19 patients and samples, Mean (SD) (score range; 1–5)	3.09 (1.30)	2.59 (1.19)	3.59 (1.20)	*<0.001*
***Estimated number of*** ***confirmed or suspected COVID-19 cases and samples that participants dealt with***				
Zero	227 (53.8)	160 (75.8)	67 (31.8)	*<0.001*
1–49	91 (21.6)	31 (14.7)	60 (28.4)	
50–100	40 (9.5)	9 (4.3)	31 (14.7)	
>100	64 (15.2)	11 (5.2)	53 (25.1)	
***Receiving an exceptional education to deal with COVID-19 patients***				
No	293 (69.4)	152 (72.0)	141 (66.8)	*0.245*
Yes	129 (30.6)	59 (28.0)	70 (33.2)	
***Participants’ evaluations of their institution preparedness to deal with COVID-19 patients***				
Very bad	26 (6.2)	9 (4.3)	17 (8.1)	*0.002*
Bad	63 (14.9)	25 (11.8)	38 (18.0)	
Fair	98 (23.2)	38 (18.0)	60 (28.4)	
Good	116 (27.5)	65 (30.8)	51 (24.2)	
Very good	92 (21.8)	57 (27.0)	35 (16.6)	
Excellent	27 (6.4)	17 (8.1)	10 (4.7)	
***Perceived changes in work schedule and intensity due to COVID-19 pandemic***				
No perceived changes	27 (6.4)	10 (4.7)	17 (8.1)	*0.474*
A little	31 (7.3)	19 (9.0)	12 (5.7)	
Some	78 (18.5)	39 (18.5)	39 (18.5)	
Much	165 (39.1)	81 (38.4)	84 (39.8)	
Very much	121 (28.7)	62 (29.4)	59 (28.0)	

The Chi-square test assessed the differences between onset and one-year samples for socio-demographic and occupational characteristics. The Student’s t-test was used to estimate the difference in means of perceived level of contact with COVID-19 patients between the two groups; ^®^ Age was defined as a categorical variable with four groups, divided approximately at the interquartile ranges; * Unmarried category included single (never married), widowed, and divorced participants; ^†^ Others included nurses, pharmacists, and technicians; ^¥^ COVID-19 vaccine was not available at the onset of the COVID-19 pandemic.

**Table 2:** Scores and severity categories of anxiety in the total cohort and the subgroups.

Characteristic	Total Cohort, *n* = 422	Onset Group, *n* = 211	One-Year Group, *n* = 211	*p*-Value
***GAD-7, anxiety***				
Total score, Mean (SD)	7.05 (4.83)	6.15 (4.15)	7.94 (5.29)	*<0.001*
**Anxiety severity categories, *n* (%)**				
Normal	132 (31.3)	74 (35.1)	58 (27.5)	*0.003*
Mild	190 (45.0)	102 (48.3)	88 (41.7)	
Moderate	58 (13.7)	24 (11.4)	34 (16.1)	
Severe	42 (10.0)	11 (5.2)	31 (14.7)	

One-way ANOVA was used to estimate the difference in means of GAD-7 scores, while the chi-square test was conducted to assess the differences in anxiety severity categories; Abbreviations: GAD-7, 7-item Generalized Anxiety Disorder.

**Table 3:** Differences in the scores and severity categories of anxiety among the onset group (*n* = 211).

Characteristic	*GAD-7, Anxiety*						
	Total Score, Mean (SD)	*p*-Value	*Anxiety Severity Categories, n (%)*				
			Normal	Mild	Moderate	Severe	*p*-Value
***Age, y***							
23–27	7.49 (4.51)	*0.015*	13 (27.7)	20 (42.6)	9 (19.1)	5 (10.6)	*0.005*
28–31	6.09 (4.06)		18 (32.1)	30 (53.6)	5 (8.9)	3 (5.4)	
32–39	6.29 (4.25)		22 (37.3)	28 (47.5)	6 (10.2)	3 (5.1)	
≥40	4.78 (3.38)		21 (42.9)	24 (49.0)	4 (8.2)	0 (0.0)	
***Gender***							
Male	5.44 (4.04)	*<0.001*	65 (42.2)	72 (46.8)	11 (7.1)	6 (3.9)	*<0.001*
Female	8.07 (3.85)		9 (15.8)	30 (52.6)	13 (22.8)	5 (8.8)	
***Marriage status***							
Unmarried *	7.19 (4.63)	*0.005*	22 (27.8)	38 (48.1)	10 (12.7)	9 (11.4)	*0.010*
Married	5.53 (3.72)		52 (39.4)	64 (48.5)	14 (10.6)	2 (1.5)	
***Living with elderly of 65 years old or older***							
No	5.91 (4.18)	*0.313*	46 (36.8)	59 (47.2)	13 (10.4)	7 (5.6)	*0.876*
Yes	6.50 (4.09)		28 (32.6)	43 (50.0)	11 (12.8)	4 (4.7)	
***Occupation***							
Physician	6.36 (4.20)	*0.174*	51 (31.1)	85 (51.8)	18 (11.0)	10 (6.1)	*0.095*
Others ^†^	5.43 (3.91)		23 (48.9)	17 (36.2)	6 (12.8)	1 (2.1)	
***Monthly income, Jordanian Dinar (JD)***							
<500	8.60 (4.74)	*<0.001*	4 (16.0)	13 (52.0)	4 (16.0)	4 (16.0)	*0.003*
500–1000	6.78 (4.16)		32 (29.9)	53 (49.5)	15 (14.0)	7 (6.5)	
1000–2000	4.21 (3.17)		20 (60.6)	11 (33.3)	2 (6.1)	0 (0.0)	
>2000	4.76 (3.37)		18 (39.1)	25 (54.3)	3 (6.5)	0 (0.0)	
***COVID-19 characteristics***							
COVID-19 tested	7.63 (4.59)	*0.004*	12 (24.5)	22 (44.9)	9 (18.4)	6 (12.2)	*0.012*
Direct contact with COVID-19 patients and samples	7.75 (4.89)	*0.001*	11 (21.2)	24 (46.2)	10 (19.2)	7 (13.5)	*0.001*
Perceived contact with COVID-19 patients and samples, Mean (SD)			2.35 (1.22)	2.60 (1.17)	3.04 (1.08)	3.09 (1.04)	*0.037*
***Estimated number of confirmed or suspected COVID-19 cases and samples that participants dealt with***							
Zero	5.42 (3.61)	*<0.001*	63 (39.4)	80 (50.0)	15 (9.4)	2 (1.3)	*<0.001*
1–49	7.61 (4.57)		6 (19.4)	16 (51.6)	6 (19.4)	3 (9.7)	
50–100	9.44 (3.81)		1 (11.1)	4 (44.4)	3 (33.3)	1 (11.1)	
>100	10.00 (6.26)		2 (18.2)	1 (9.1)	3 (27.3)	5 (45.5)	
***Receiving an exceptional education to deal with COVID-19 patients***							
No	6.74 (4.18)	*0.001*	44 (28.9)	78 (51.3)	20 (13.2)	10 (6.6)	*0.016*
Yes	4.63 (3.69)		30 (50.8)	24 (40.7)	4 (6.8)	1 (1.7)	
***Participants’ evaluations of their institution preparedness to deal with COVID-19 patients***							
Very bad	9.78 (3.07)	*<0.001*	0 (0.0)	3 (33.3)	5 (55.6)	1 (11.1)	*<0.001*
Bad	8.68 (5.51)		7 (28.0)	9 (36.0)	4 (16.0)	5 (20.0)	
Fair	6.82 (3.84)		10 (26.3)	22 (57.9)	4 (10.5)	2 (5.3)	
Good	5.69 (3.41)		22 (33.8)	37 (56.9)	5 (7.7)	1 (1.5)	
Very good	5.39 (3.64)		24 (42.1)	26 (45.6)	6 (10.5)	1 (1.8)	
Excellent	3.35 (4.34)		11 (64.7)	5 (29.4)	0 (0.0)	1 (5.9)	
***Perceived changes in work schedule and intensity due to COVID-19 pandemic***							
No changes	5.80 (3.88)	*0.151*	3 (30.0)	6 (60.0)	1 (10.0)	0 (0.0)	*0.276*
A little	5.89 (5.43)		9 (47.4)	8 (42.1)	0 (0.0)	2 (10.5)	
Some	4.85 (3.54)		19 (48.7)	17 (43.6)	2 (5.1)	1 (2.6)	
Much	6.22 (3.81)		25 (30.9)	43 (53.1)	9 (11.1)	4 (4.9)	
Very much	7.02 (4.42)		18 (29.0)	28 (45.2)	12 (19.4)	4 (6.5)	

Student’s t-test or one-way ANOVA was conducted to investigate the differences in the GAD-7 scale scores with socio-demographic and occupational characteristics. In contrast, the differences in the severity categories of anxiety were assessed using a chi-square test; * Unmarried category included single, widowed, and divorced participants; ^†^ Others included nurses, pharmacists, and technicians.

**Table 4:** Differences in the GAD-7 scores and anxiety severity categories among the one-year group (*n* = 211).

Characteristic	*GAD-7*, *Anxiety*						
	Total Score, Mean (SD)	*p*-Value	*Anxiety Severity Categories, n* (%)				
			Normal	Mild	Moderate	Severe	*p*-Value
***Age, y***							
23–27	9.65 (5.23)	*<0.001*	8 (18.6)	15 (34.9)	8 (18.6)	12 (27.9)	*0.016*
28–31	8.96 (5.38)		10 (19.6)	22 (43.1)	10 (19.6)	9 (17.6)	
32–39	8.17 (5.09)		15 (25.4)	26 (44.1)	11 (18.6)	7 (11.9)	
≥40	5.53 (4.71)		25 (43.1)	25 (43.1)	5 (8.6)	3 (5.2)	
***Gender***							
Male	7.02 (5.12)	*<0.001*	52 (35.4)	60 (40.8)	20 (13.6)	15 (10.2)	*<0.001*
Female	10.05 (5.11)		6 (9.4)	28 (43.8)	14 (21.9)	16 (25.0)	
***Marriage status***							
Unmarried *	9.24 (5.07)	*0.002*	15 (16.9)	36 (40.4)	21 (23.6)	17 (19.1)	*0.003*
Married	6.99 (5.27)		43 (35.2)	52 (42.6)	13 (10.7)	14 (11.5)	
***Living with elderly of 65 years old or older***							
No	8.23 (5.31)	*0.486*	25 (27.2)	37 (40.2)	14 (15.2)	16 (17.4)	*0.805*
Yes	7.71 (5.29)		33 (27.7)	51 (42.9)	20 (16.8)	15 (12.6)	
***Occupation***							
Physician	8.37 (5.43)	*0.004*	44 (24.4)	75 (41.7)	31 (17.2)	30 (16.7)	*0.004*
Others ^†^	5.45 (3.54)		14 (45.2)	13 (41.9)	3 (9.7)	1 (3.2)	
***Monthly income, Jordanian Dinar (JD)***							
<500	12.77 (5.08)	*<0.001*	2 (6.5)	5 (16.1)	12 (38.7)	12 (38.7)	*<0.001*
500–1000	8.54 (5.03)		16 (19.5)	39 (47.6)	13 (15.9)	14 (17.1)	
1000–2000	7.18 (5.13)		10 (30.3)	16 (48.5)	4 (12.1)	3 (9.1)	
>2000	5.26 (3.91)		30 (46.2)	28 (43.1)	5 (7.7)	2 (3.1)	
***COVID-19 characteristics***							
Vaccinated	7.75 (5.32)	*0.745*	36 (23.8)	69 (45.7)	24 (15.9)	22 (14.6)	*0.202*
COVID-19 tested	8.31 (5.24)	*0.007*	42 (22.8)	81 (44.0)	32 (17.4)	29 (15.8)	*0.001*
COVID-19 infected	8.82 (5.65)	*0.024*	24 (24.7)	33 (34.0)	21 (21.6)	19 (19.6)	*0.025*
Direct contact with COVID-19 patients and samples	8.83 (5.47)	*<0.001*	32 (22.1)	59 (40.7)	25 (17.2)	29 (20.0)	*0.002*
Perceived contact with COVID-19 patients and samples, Mean (SD)			3.07 (1.29)	3.53 (1.16)	4.06 (1.01)	4.19 (0.87)	*<0.001*
***Estimated number of confirmed or suspected COVID-19 cases and samples that participants were dealt with***							
Zero	6.21 (4.57)	*<0.001*	26 (38.8)	27 (40.3)	12 (17.9)	2 (3.0)	*<0.001*
1–49	7.32 (4.72)		13 (21.7)	36 (60.0)	4 (6.7)	7 (11.7)	
50–100	9.16 (5.48)		8 (25.8)	9 (29.0)	9 (29.0)	5 (16.1)	
>100	10.11 (5.83)		11 (20.8)	16 (30.2)	9 (17.0)	17 (32.1)	
***Receiving an exceptional education to deal with COVID-19 patients***							
No	9.10 (5.49)	*<0.001*	29 (20.6)	55 (39.0)	31 (22.0)	26 (18.4)	*<0.001*
Yes	5.60 (3.97)		29 (41.4)	33 (47.1)	3 (4.3)	5 (7.1)	
***Participants’ evaluations of their institution preparedness to deal with COVID-19 patients***							
Very bad	14.29 (4.78)	*<0.001*	0 (0.0)	4 (23.5)	3 (17.6)	10 (58.8)	*<0.001*
Bad	9.39 (5.68)		8 (21.1)	13 (34.2)	9 (23.7)	8 (21.1)	
Fair	7.58 (4.20)		13 (21.7)	31 (51.7)	12 (20.0)	4 (6.7)	
Good	6.78 (4.80)		17 (33.3)	23 (45.1)	8 (15.7)	3 (5.9)	
Very good	6.14 (5.25)		14 (40.0)	15 (42.9)	1 (2.9)	5 (14.3)	
Excellent	5.90 (4.68)		6 (60.0)	2 (20.0)	1 (10.0)	1 (10.0)	
***Perceived changes in work schedule and intensity due to COVID-19 pandemic***							
No changes	5.65 (3.45)	*<0.001*	9 (52.9)	5 (29.4)	3 (17.6)	0 (0.0)	*<0.001*
A little	6.33 (4.89)		3 (25.0)	8 (66.7)	0 (0.0)	1 (8.3)	
Some	6.23 (4.77)		15 (38.5)	19 (48.7)	1 (2.6)	4 (10.3)	
Much	7.71 (4.75)		20 (23.8)	37 (44.0)	20 (23.8)	7 (8.3)	
Very much	10.37 (6.02)		11 (18.6)	19 (32.2)	10 (16.9)	19 (32.2)	

Student’s t-test or one-way ANOVA was conducted to investigate the differences in the GAD-7 scale scores with socio-demographic and occupational characteristics. In contrast, the differences in the severity categories of anxiety were assessed using a chi-square test. * Unmarried category included single, widowed, and divorced participants. ^†^ Others included nurses, pharmacists, and technicians.

**Table 5:** Risk factors for probable Generalized Anxiety Disorder (GAD) diagnosis among healthcare workers identified by binary logistic regression analyses *.

Variable	No. of Disease Cases/ No. of Total Cases (%)	Adjusted OR	95% CI (Lower–Upper)	*p*-Value
***Onset sample (n = 211)***				
***Gender***				
Male	17/154 (11.0)	REF	REF	*REF*
Female	18/57 (31.6)	3.224	1.440–7.218	*0.004*
***Monthly income, Jordanian Dinar (JD)***	–	0.690	−0.094–−1.335	*0.028*
***COVID-19 test***				
Yes	15/49 (30.6)	2.196	0.855–5.637	*0.102*
No	20/162 (12.3)	REF	REF	*REF*
***Direct contact with COVID-19 patients and samples***				
Yes	17/52 (32.7)	2.292	0.909–5.777	*0.079*
No	18/159 (11.3)	REF	REF	*REF*
***Receiving an exceptional education to deal with COVID-19 patients***				
Yes	5/59 (8.5)	REF	REF	*REF*
No	30/152 (19.7)	3.245	1.123–9.378	*0.030*
***Participants’ evaluations of institution preparedness to deal with COVID-19 patients***				
Very bad	6/9 (66.7)	7.075	0.477–104.834	*0.155*
Bad	9/25 (36.0)	2.861	0.266–30.800	*0.386*
Fair	6/38 (15.8)	1.247	0.121–12.828	*0.853*
Good	6/65 (9.2)	0.660	0.064–6.829	*0.728*
Very good	7/57 (12.3)	1.477	0.160–13.661	*0.731*
Excellent	1/17 (5.9)	REF	REF	*REF*
***One-year sample (n = 211) ^†^***				
***Gender***				
Male	35/147 (23.8)	REF	REF	*REF*
Female	30/64 (46.9)	1.888	0.847–4.206	*0.120*
***Occupation***				
Physician	61/180 (33.9)	3.214	0.868–11.899	*0.080*
Others	4/31 (12.9)	REF	REF	*REF*
***Monthly income, Jordanian Dinar (JD)***				
<500	24/31 (77.4)	12.945	3.537–47.380	*<0.001*
500–1000	27/82 (32.9)	3.273	1.197–8.949	*0.021*
1000–2000	7/33 (21.2)	1.611	0.452–5.738	*0.462*
>2000	7/65 (10.8)	REF	REF	*REF*
***Hx of COVID-19 infection***				
Yes	40/97 (41.2)	1.707	0.790–3.688	*0.174*
No	25/114 (21.9)	REF	REF	*REF*
***Direct contact with COVID-19 patients and samples***				
Yes	54/145 (37.2)	2.238	0.944–5.302	*0.067*
No	11/66 (16.7)	REF	REF	*REF*
***Receiving an exceptional education to deal with COVID-19 patients***				
Yes	8/70 (11.4)	REF	REF	*REF*
No	57/141 (40.4)	6.052	2.394–15.296	*<0.001*
***Participants’ evaluations of institution preparedness to deal with COVID-19 patients***				
Very bad	13/17 (76.5)	8.716	1.215–62.523	*0.031*
Bad	17/38 (44.7)	2.694	0.483–15.024	*0.258*
Fair	16/60 (26.7)	1.204	0.221–6.563	*0.830*
Good	11/51 (21.6)	0.912	0.161–5.169	*0.917*
Very good	6/35 (17.1)	1.028	0.166–6.379	*0.977*
Excellent	2/10 (20.0)	REF	REF	*REF*

* Socio-demographic characteristics (including age, gender, marriage status, living with elderly, occupation, and monthly income), COVID-19 characteristics (including previous COVID-19 testing and direct contact with COVID-19 patients or samples), receiving an exceptional education to deal with COVID-19 patients, participants’ evaluations of institution preparedness, and perceived changes in work schedule and intensity due to COVID-19 pandemic were included as independent explanatory variables in the backward stepwise binary logistic regression model; ^†^ Vaccination status and previous COVID-19 infection were included as independent explanatory variables for GAD risk factors in the one-year group analysis, while these were not included in the onset group analysis.

## Data Availability

Some or all data generated during the study are proprietary or confidential in nature and may only be provided with restrictions. The datasets generated and analyzed during the current study are available with the corresponding author.
